# Unmasking paramagnetic rim multiple sclerosis lesions: the advantages of quantitative susceptibility mapping over phase imaging

**DOI:** 10.1093/braincomms/fcaf037

**Published:** 2025-01-27

**Authors:** Àlex Rovira, Deborah Pareto

**Affiliations:** Section of Neuroradiology, Department of Radiology (IDI), Hospital Universitari Vall d’Hebron, Universitat Autònoma de Barcelona, Barcelona 08035, Spain; Section of Neuroradiology, Department of Radiology (IDI), Hospital Universitari Vall d’Hebron, Universitat Autònoma de Barcelona, Barcelona 08035, Spain

## Abstract

This scientific commentary refers to ‘Quantitative susceptibility mapping is more sensitive and specific than phase imaging in detecting chronic active multiple sclerosis lesion rims: pathological validation’, by Gillen *et al*. (https://doi.org/10.1093/braincomms/fcaf011).

**This scientific commentary refers to ‘Quantitative susceptibility mapping is more sensitive and specific than phase imaging in detecting chronic active multiple sclerosis lesion rims: pathological validation’, by Gillen *et al*. (** https://doi.org/10.1093/braincomms/fcaf011 **).**

Multiple sclerosis is a chronic condition of the CNS driven by immune-mediated mechanisms. It is marked by pathological processes such as inflammation, demyelination, axonal damage, and astrogliosis. These changes result in both localized and widespread CNS abnormalities, culminating in the gradual accumulation of physical and cognitive impairments over time.

Advances in the understanding of multiple sclerosis immunopathology have led to the development of a diverse array of disease-modifying therapies (DMTs) that specifically target the adaptive immune system’s role in the disease. These treatments have revolutionized multiple sclerosis management, as evidenced by a significant reduction in annual relapse rates and a marked increase in MRI studies showing no new T_2_ or gadolinium-enhancing lesions, which are widely accepted surrogate markers of disease activity.

Despite these significant advancements in multiple sclerosis treatment, the disease remains incurable, with many patients continuing to experience progressive disability. This highlights the limitations of current DMTs, which may not fully prevent the long-term accumulation of disability.

The disconnection between the inflammatory mechanisms driving relapses and those responsible for progressive disability is encapsulated in the concept of progression independent of relapse activity (PIRA). PIRA is predominantly attributed to the ‘smouldering’ component of multiple sclerosis, characterized by chronic low-grade inflammation and neurodegeneration that persist even in the absence of clinical relapses or new MRI-detectable lesions.^1^ This smouldering pathology is believed to result from the activation of innate immune responses within the CNS. A hallmark of this process is the accumulation of iron-laden macrophages and activated microglia at the edges of chronic active lesions (CALs), which are visible as paramagnetic rim lesions (PRLs) on susceptibility-sensitive MRI sequences.^2^

These immune cells release proinflammatory cytokines and other inflammatory mediators, which impair tissue repair and drive ongoing damage to the surrounding CNS tissue. This process results in the gradual, concentric expansion of CALs, termed slowly evolving lesions.^3^ The lack of effective repair and remyelination within these lesions is evidenced on MRI by progressive changes in imaging biomarkers, including reduced magnetization transfer ratio, increased radial diffusivity and prolonged T_1_ and T_2_ relaxation times.^4,5^ These changes reflect ongoing demyelination, axonal loss and disruption of tissue structure within the lesions, underscoring the destructive nature of this chronic inflammatory process.

The detection of PRLs has gained significant attention in recent years, not only because they are considered a key biomarker of the smouldering component of multiple sclerosis but also due to their potential as a tool for evaluating the efficacy of emerging DMTs targeting innate immune cells, such as Bruton tyrosine kinase inhibitors. Furthermore, PRLs have also gained prominence as a diagnostic marker in specific clinical scenarios, as highlighted in the 2024 revision of the McDonald diagnostic criteria.^6^ In these updated criteria, the identification of at least one PRL is considered an additional criterion that can aid in establishing a diagnosis of multiple sclerosis in cases where conventional MRI findings are inconclusive. This highlights the growing recognition of PRLs as a valuable tool in refining diagnostic precision.

Many studies investigating PRLs have utilized 7T MRI scanners, which are not commonly available in clinical practice. However, growing evidence indicates that PRLs can also be reliably detected using 3T and 1.5T MRI systems, provided that appropriate susceptibility-sensitive sequences are employed.

A recent systematic review and meta-analysis reported a composite specificity of 98% for PRLs in diagnosing multiple sclerosis, with sensitivity ranging from 10 to 92.3%.^2^ The variability in sensitivity is largely influenced by the imaging techniques employed, emphasizing the importance of optimizing imaging methodologies to ensure accurate detection and highlighting the need to identify the most effective and reliable MRI technique for detecting PRLs within a reasonable scanning time.

Several susceptibility-based brain MRI sequences and techniques have been employed to identify PRLs, including T_2_*- and R_2_*-weighted magnitude imaging, phase imaging, susceptibility-weighted imaging and quantitative susceptibility mapping (QSM). All these methods are highly sensitive to the magnetic properties of tissues, capturing the distribution of both paramagnetic substances, such as iron, and diamagnetic substances, like myelin.^7^ However, each of these techniques is subject to various limitations, influenced by factors such as acquisition times, accuracy, availability, and acquisition and post-processing complexity. Among these, phase imaging emerges as a relatively accurate and widely available technique for clinical practice, despite its well-known disadvantages, such as the influence of non-local effects of magnetic susceptibility on phase changes, which can compromise the accuracy of PRL detection.^7^

On the other hand, QSM is an advanced post-processing method that quantifies the magnetic susceptibility distribution within tissues, allowing for detailed visualization of substances like iron and myelin. In the context of multiple sclerosis, these components undergo substantial alterations within lesions, exhibiting magnetic susceptibility values that differ significantly from those of normal brain parenchyma.

In this issue of *Brain Communications*, Gillen *et al*.^8^ present the first *ex vivo* validation of both phase imaging and QSM for detecting PRLs using post-mortem MRI. Their study reveals that while both techniques demonstrate similarly high sensitivity (>90%) for detecting PRLs, QSM outperforms phase imaging in terms of sensitivity and positive predictive value.

This distinction can be attributed to the physics underlying phase imaging, where susceptibility distributions can create rim-like artefacts, resulting in false-positive PRL detections and consequently leading to an overestimation of PRLs. QSM addresses this limitation by employing dipole deconvolution to recover local tissue susceptibility, providing a more accurate representation of underlying pathology.

These findings position QSM as a more reliable and precise marker for identifying PRLs in multiple sclerosis lesions compared to phase imaging, highlighting its potential to become the preferred MRI method for detecting this type of chronic multiple sclerosis lesions. Moreover, the authors emphasize an additional advantage of QSM: its capacity to quantitatively measure iron content. This capability establishes QSM as a biomarker for the smouldering component of multiple sclerosis, which can be used longitudinally to track disease progression and evaluating therapeutic interventions, offering valuable insights for both clinical and research applications.

Although the study of Gillen *et al*.^8^ provides compelling evidence of the higher accuracy of QSM over phase imaging in detecting PRLs, several challenges must be addressed to facilitate its implementation in clinical practice. First, the complexity of the QSM pipeline introduces potential sources of error, compounded by the lack of universally accepted protocols for data acquisition and post-processing, which can lead to inconsistencies between centres. Additionally, current QSM techniques are not widely available on clinical scanners and are computationally intensive, requiring advanced processing capabilities that may delay results in clinical workflows. Moreover, the performance of QSM on 1.5T scanners remains unvalidated, limiting its applicability in many clinical settings. Its integration into routine practice is further hindered by a lack of familiarity among (neuro)radiologists, emphasizing the need for dedicated training to ensure proper image interpretation.

Finally, quantitatively assessing iron distribution in multiple sclerosis lesions using QSM remains challenging due to the simultaneous contributions of both paramagnetic (e.g. iron) and diamagnetic (e.g. myelin) materials to the frequency shift and transverse relaxation of MRI signals.^9^ As a result, interpreting susceptibility shifts must be done with caution, as QSM cannot separate the opposing effects of increased paramagnetic iron and decreased diamagnetic myelin on the signal. This limitation can be addressed by combining QSM with myelin-specific imaging biomarkers^9^ or by employing models capable of separating these susceptibility sources, such as χ-separation imaging.^10^ However, the incorporation of these additional techniques into clinical MRI protocols may not be feasible.

Overcoming these challenges will require the development of standardized acquisition and processing protocols, advancements in computational algorithms for faster and more robust QSM methods and targeted educational initiatives to familiarize (neuro)radiologists with the technique’s benefits, limitations and interpretation. While QSM holds significant potential for clinical applications, addressing these barriers is essential for its widespread adoption in clinical practice.
